# Limiting viral replication in hepatocytes alters Rift Valley fever virus disease manifestations

**DOI:** 10.1128/jvi.00853-23

**Published:** 2023-09-28

**Authors:** Lingqing Xu, Alden C. Paine, Dominique J. Barbeau, Frances Alencastro, Andrew W. Duncan, Anita K. McElroy

**Affiliations:** 1 Department of Pediatrics, Division of Pediatric Infectious Disease, University of Pittsburgh School of Medicine, Pittsburgh, Pennsylvania, USA; 2 Center for Vaccine Research, University of Pittsburgh, Pittsburgh, Pennsylvania, USA; 3 Department of Pathology, McGowan Institute for Regenerative Medicine, Pittsburgh Liver Research Center, UPMC Hillman Cancer Center, University of Pittsburgh, Pittsburgh, Pennsylvania, USA; 4 Department of Bioengineering, University of Pittsburgh, Pittsburgh, Pennsylvania, USA; Loyola University Chicago, Maywood, Illinois, USA

**Keywords:** Rift Valley fever virus, viral pathogenesis, hepatitis, encephalitis

## Abstract

Rift Valley fever virus (RVFV) causes mild to severe disease in humans and livestock. Outbreaks of RVFV have been reported throughout Africa and have spread outside Africa since 2000, calling for urgent worldwide attention to this emerging virus. RVFV directly infects the liver, and elevated transaminases are a hallmark of severe RVFV infection. However, the specific contribution of viral replication in hepatocytes to pathogenesis of RVFV remains undefined. To address this, we generated a recombinant miRNA-targeted virus, RVFVmiR-122, to limit hepatocellular replication. MicroRNAs are evolutionarily conserved non-coding RNAs that regulate mRNA expression by targeting them for degradation. RVFVmiR-122 includes an insertion of four target sequences of the liver-specific miR-122. In contrast to control RVFVmiR-184, which contains four target sequences of mosquito-specific miR-184, RVFVmiR-122 has restricted replication *in vitro* in primary mouse hepatocytes. RVFVmiR-122-infected C57BL/6 mice survived acute hepatitis and instead developed late-onset encephalitis. This difference in clinical outcome was eliminated in *Mir-122 KO* mice, confirming the specificity of the finding. Interestingly, C57BL/6 mice infected with higher doses of RVFVmiR-122 had a higher survival rate which was correlated with faster clearance of virus from the liver, suggesting a role for activation of host immunity in the phenotype. Together, our data demonstrate that miR-122 can specifically restrict the replication of RVFVmiR-122 in liver tissue both *in vitro* and *in vivo*, and this restriction alters the clinical course of disease following RVFVmiR-122 infection.

**IMPORTANCE:**

Rift Valley fever virus (RVFV) is a hemorrhagic fever virus that causes outbreaks in humans and livestock throughout Africa and has spread to continents outside Africa since 2000. However, no commercial vaccine or treatment is currently available for human use against RVFV. Although the liver has been demonstrated as a key target of RVFV, the contribution of viral replication in hepatocytes to overall RVFV pathogenesis is less well defined. In this study we addressed this question by using a recombinant miRNA-targeted virus with restricted replication in hepatocytes. We gained a better understanding of how this individual cell type contributes to the development of disease caused by RVFV. Techniques used in this study provide an innovative tool to the RVFV field that could be applied to study the consequences of limited RVFV replication in other target cells.

## INTRODUCTION

Rift Valley fever virus (RVFV) is a negative-sense, single-stranded RNA virus that belongs to the *Phlebovirus* genus under the *Bunyavirales* order in the *Phenuiviridae* family (1). It is an important emerging arbovirus that causes Rift Valley fever, a hemorrhagic fever that threatens humans and livestock in many parts of sub-Saharan Africa and the Middle East (2). It was first identified in 1931 during an epizootic in sheep on a farm in the Rift Valley of Kenya (3). During 1999–2021, more than 1,100 events of human or animal RVFV transmission were reported in 39 countries, with an expansion in both range and frequency (4).

Transmitted by mosquitoes or the bodily fluids of infected animals, RVFV can cause severe disease in both humans and livestock. Pregnant ruminants are subject to a high rate of abortion, whereas newborn lambs die of acute hepatitis (5). Most infected humans develop a self-limiting febrile illness showing mild symptoms of headache, fever, malaise, and myalgia; however, some individuals develop severe disease including hepatitis, hemorrhagic fever, ocular disease, or encephalitis, often with high levels of mortality (6 – 8). Due to its ability to cause severe disease in humans and livestock during epidemics and epizootics, and the risk of global spread, RVFV has been listed as a priority pathogen by the World Health Organization since 2017 (9). It is also listed as a category A priority pathogen by the National Institute of Allergy and Infectious Diseases (NIAID) and classified as a select agent by both Department of Health and Human Services and United States Department of Agriculture. Unfortunately, there are currently no licensed vaccines or treatments for human use against RVFV, necessitating the urgency to better understand the pathogenesis of this virus (10).

Previous studies have established that three cell types represent the major targets of RVFV *in vivo*: hepatocytes, neurons, and mononuclear phagocytic cells (11, 12). However, the contribution of infection of each cell type to viral pathogenesis has not been defined. In this study, we focus on the role of hepatocyte tropism in the pathogenesis of RVFV. The liver is a major site of RVFV replication, and liver damage can contribute to jaundice and hemorrhagic disease (7, 12). Patients who died from RVFV infection had much higher levels of biomarkers of hepatocyte damage than those who survived (13). To study the contribution of viral replication in hepatocytes to RVFV pathogenesis, we took advantage of tissue-specific miRNAs to restrict viral replication in cells of interest (14 – 16).

MicroRNAs are short non-coding RNAs of ~22 nucleotides that play key roles in essential developmental and physiologic processes (17). They achieve this by regulating mRNA expression via guiding Argonaute proteins to target mRNAs, which leads to translation repression and mRNA decay (18). Most miRNAs are evolutionarily conserved in related species, and many have expression patterns that are cell type-specific (19, 20). To study the role of liver-specific replication on the pathogenesis of RVFV, we generated a recombinant RVFVmiR-122 with an insertion of four repeats of sequences targeted by miR-122, a miRNA highly expressed in hepatocytes, into the genome of the RVFV S segment (19, 21). The viral S segment of RVFV encodes two proteins in an ambisense manner: nucleoprotein N, which is required for transcription and replication of viral RNA, and a non-structural protein NSs, which is a major virulence factor in RVFV infection (22, 23). The genome of RVFV also contains a large (L) and a medium (M) segment. The L segment encodes the RNA-dependent RNA polymerase (24). The M segment encodes two surface glycoproteins, Gn and Gc, and two non-structural proteins, NSm and the 78 kDa protein (25). Additionally, we generated RVFVmiR-184 with an insertion of sequences targeted by miR-184, a mosquito-specific miRNA, as a control (26).

By comparing these two miRNA-targeted recombinant RVFV *in vitro* and *in vivo*, we characterized the consequences of hepatocyte-specific restriction of viral replication and investigated the role of host immunity in the process. This study demonstrates that restricting viral replication in hepatocytes changes the disease manifestation of RVFV infection in C57BL/6 mice, indicating that viral replication in hepatocytes is a key determinant of RVFV pathogenesis.

## RESULTS

### Recombinant RVFVmiR-122 shows restricted replication in primary mouse hepatocytes

**Fig 1:**
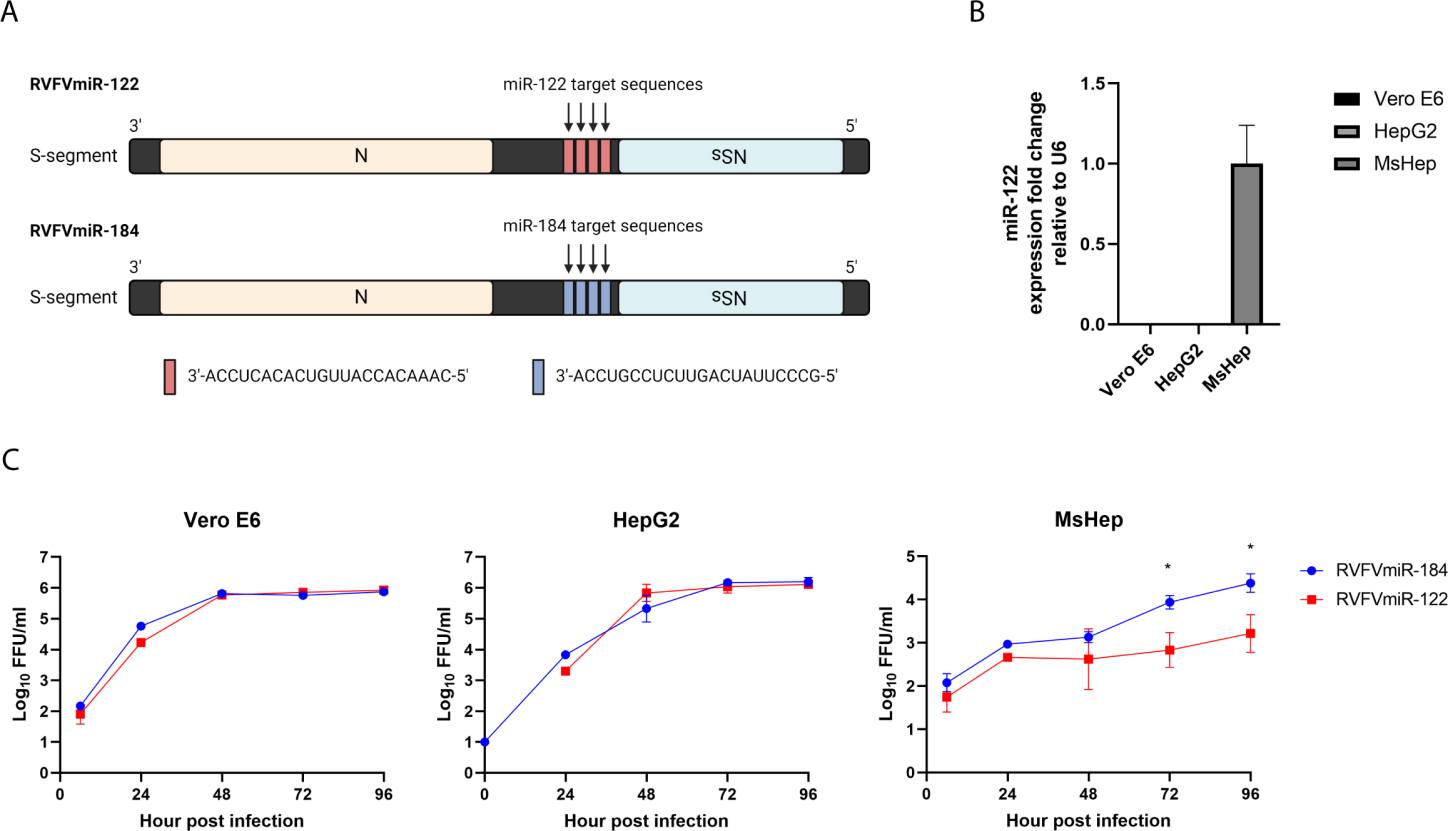
RVFVmiR-122 showed restricted replication in primary mouse hepatocytes compared to control RVFVmiR-184. (**A**) Schematic design of RVFVmiR-122 and RVFVmiR-184. Image was created in BioRender. (**B**) RNA extracted from triplicates of Vero E6 or HepG2 cells or primary mouse hepatocytes (MsHep) was used for reverse transcription followed by real-time PCR targeting either miR-122 or U6 gene as control. Fold expression change of miR-122 relative to U6 in each cell line was normalized to that of MsHep. (**C**) Vero E6, HepG2, or primary mouse hepatocytes were infected with RVFVmiR-122 or RVFVmiR-184 at MOI (multiplicity of infection) of 0.1. Supernatants were collected at various time points post-infection and analyzed by focus forming unit (FFU) assay. Unpaired *t*-test was used to compare data set at 72 h and 96 h. *P < 0.05.

To study the role of viral replication in hepatocytes on RVFV pathogenesis, we generated RVFVmiR-122 with an insertion of four repeats of sequences targeted by miR-122, a miRNA highly expressed in hepatocytes, into the genome strand of the RVFV S segment after the coding region of the NSs gene (Fig. 1A). We also generated RVFVmiR-184 with an insertion of sequences targeted by miR-184, a mosquito-specific miRNA, as a control (Fig. 1A). To examine the viral replication of these two viruses *in vitro*, we infected Vero E6 cells, HepG2 cells, or primary mouse hepatocytes with either RVFVmiR-122 or RVFVmiR-184 and collected supernatants at various times post-infection. As expected, these two viruses had no difference in replication in Vero E6 or HepG2, two cell lines that do not express miR-122 (Fig. 1B). However, RVFVmiR-122 had restricted replication compared to RVFVmiR-184 in primary mouse hepatocytes, which do express miR-122 (Fig. 1B). This was most notable at 72 h and 96 h post-infection (Fig. 1C). These data demonstrated restricted replication of RVFVmiR-122 in cells expressing miR-122.

### Disease manifestation changed from acute hepatitis to late-onset encephalitis in RVFVmiR-122-infected mice

**Fig 2:**
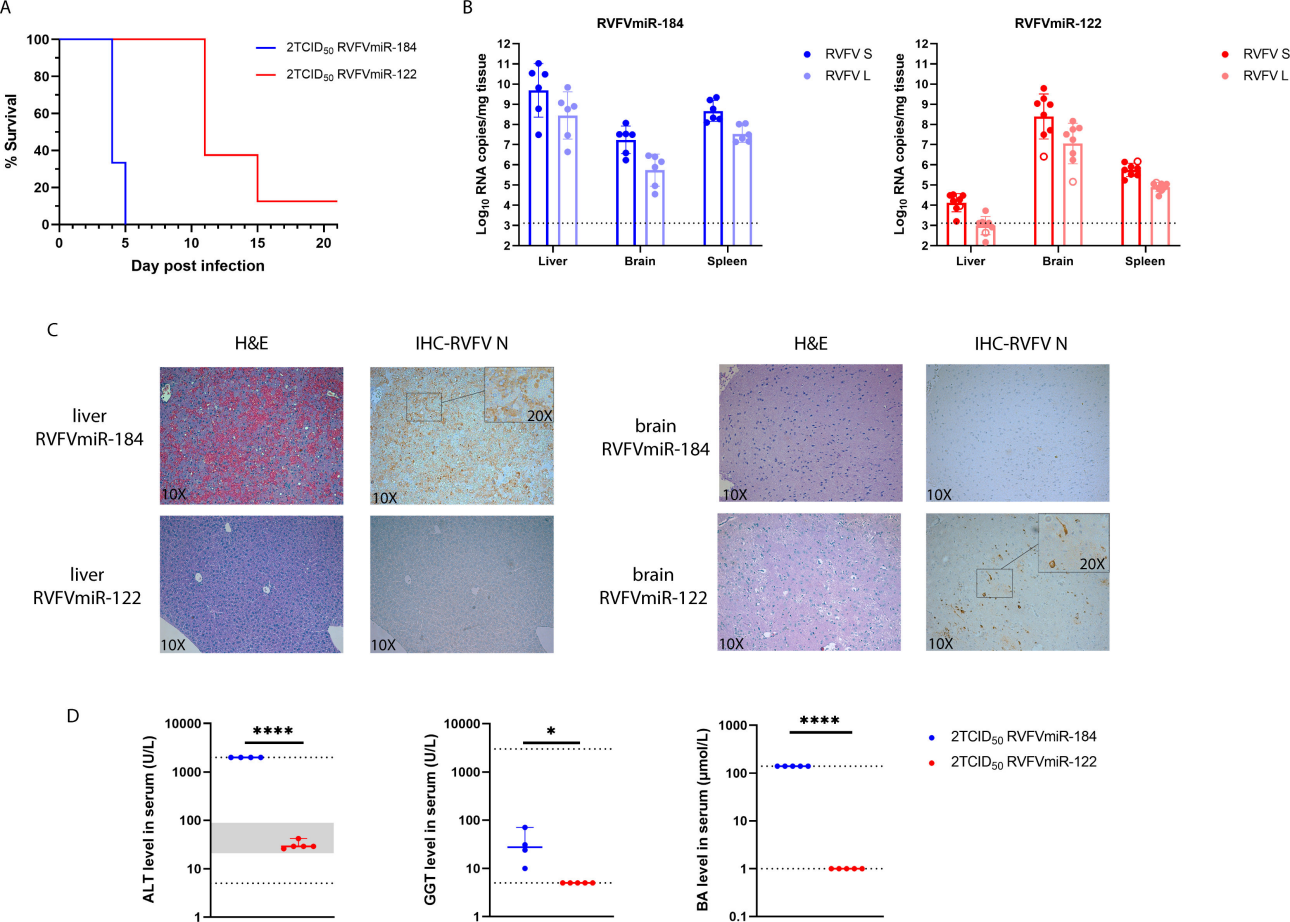
Disease manifestation changes from acute hepatitis to late-onset encephalitis in C57BL/6 mice infected with RVFVmiR-122. (**A**) C57BL/6 mice were monitored for survival after footpad infection with either RVFVmiR-184 (*n* = 6) or RVFVmiR-122 (*n* = 8). (**B**) Viral RNA loads of L or S segment of RVFV in the liver, spleen, and brain tissues from mice at the time of terminal euthanasia. Open circle in red represents one mouse that survived until the end of the experiment (21 dpi). Dotted line indicates LOD of qRT-PCR. (**C**) Representatives of liver and brain tissue staining by H&E or IHC probing for RVFV N protein imaged with 10× objective. Insets of indicated areas were imaged with 20× objective. (**D**) Blood from a subset of mice infected with either RVFVmiR-184 (*n* = 5) or RVFVmiR-122 (*n* = 5) were used for chemistry analysis at the time of euthanasia. ALT, alanine transaminase; GGT, gamma-glutamyl transferase; BA, bile acid. Dotted line represents LOD and gray area represents normal range of that test. Levels of GGT and BA in uninfected animals are at or below LOD (data not shown). Unpaired *t*-test was used for statistical analysis. *P < 0.05, ****P < 0.0001.

To examine the consequences of hepatocyte restricted replication of RVFV *in vivo*, we infected C57BL/6 mice with either RVFVmiR-184 or RVFVmiR-122 at a dose of 2 TCID_50_ (Tissue Culture Infectious Dose, 50%). Previous studies have shown that C57BL/6 mice infected with 2 TCID_50_ of wild-type (WT) RVFV (ZH501) consistently die of acute hepatitis between 3 and 4 days post-infection (dpi) (27). As expected, mice infected with control RVFVmiR-184 developed clinical symptoms and succumbed to disease early (Fig. 2A). In contrast, all mice infected with RVFVmiR-122 survived the early stage and all but one mouse developed late-onset encephalitis and required euthanasia on or after 11 dpi (Fig. 2A). Viral RNA loads in tissues of infected mice paralleled the disease timeline and manifestation. Mice infected with RVFVmiR-122 had the highest levels of viral RNA in the brain while liver viral RNA was close to the limit of detection (LOD) (Fig. 2B, red). The restricted replication appeared to be global as quantitative RT-PCR (qRT-PCR) targeting either RVFV L or RVFV S segment was similarly affected (Fig. 2B). RVFVmiR-184-infected mice had disseminated disease with high levels of viral RNA in all tissues sampled at the time of euthanasia (Fig. 2B, blue). Liver and brain tissue sections were analyzed by hematoxylin and eosin (H&E) stain and immunohistochemistry (IHC) for RVFV antigen and blood serum chemistries were assessed. RVFVmiR-184-infected mice showed hemorrhagic infiltration in the liver with diffuse viral antigen staining, as well as elevated levels of key markers of liver damage (Fig. 2C and D). In contrast, RVFVmiR-122-infected mice had normal liver histology with negative antigen staining and normal liver function at the time of euthanasia (Fig. 2C and D). Instead, RVFVmiR-122-infected mice had patches of viral antigen staining in neurons, which was consistent with high viral RNA loads observed in the brain and clinical signs of central nervous system (CNS) disease (Fig. 2C). Although there was a fair amount of viral RNA present in the brain of RVFVmiR-184-infected mice as indicated by qRT-PCR (mean = 1.73e + 07 copies per milligram tissue, RVFV L), IHC showed little RVFV antigen staining in the brain tissue. It was possible that the antigen staining was confined to certain local areas of the brain which was missed on single tissue slices or became detectable at higher viral RNA levels as in RVFVmiR-122-infected mice (mean = 2.5e + 08 copies per milligram tissue, RVFV L). Together, these data showed that C57BL/6 mice no longer develop acute hepatitis following restriction of RVFV replication in the liver, and instead develop late-onset encephalitis.

### Higher doses of RVFVmiR-122 did not cause acute hepatitis in C57BL/6 mice

**Fig 3:**
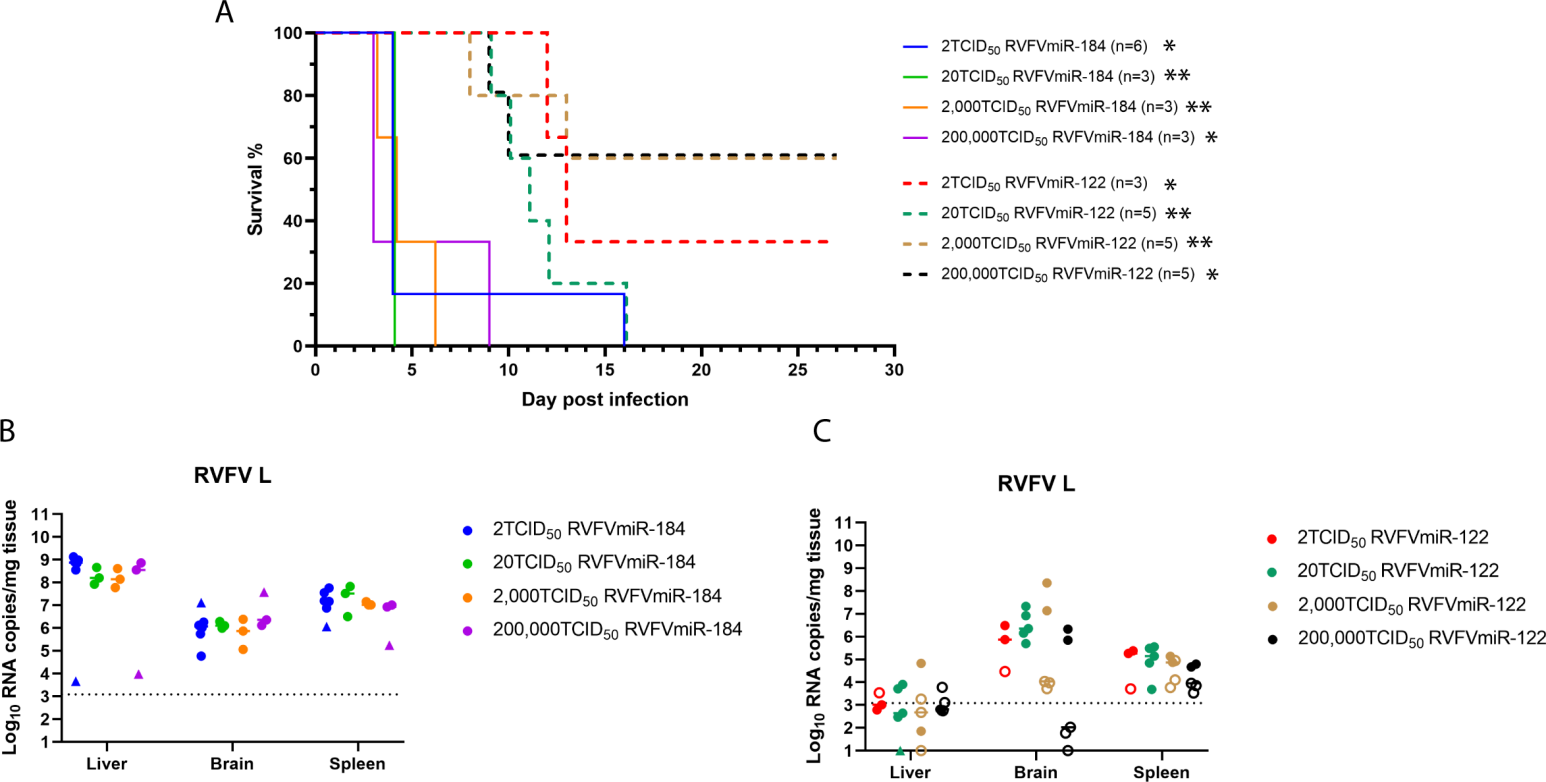
C57BL/6 mice infected with different doses of RVFVmiR-122 all survived early acute hepatitis. (**A**) C57BL/6 mice were monitored for survival after footpad infection with either RVFVmiR-184 or RVFVmiR-122 at different doses. The number of mice in each group is indicated. Gehan-Breslow-Wilcoxon test was performed for statistical analysis on RVFVmiR-184- and RVFVmiR-122-infected groups at the same dose. *P < 0.05, **P < 0.01. (**B** and **C**) Viral RNA loads in the liver, brain, and spleen tissues from mice at the time of terminal euthanasia in (**A**) after infection with RVFVmiR-184 (**B**) or RVFVmiR-122 (**C**). Solid triangles in (**B**) represent mice euthanized after 6 dpi. Open circles in (**C**) represent mice that survived until the end of the experiment (27 dpi). Dotted line indicates LOD of qRT-PCR.

We hypothesized that infecting C57BL/6 mice with increased doses of RVFVmiR-122 would eventually overwhelm the inhibitory capacity of miR-122 in the liver and change the disease manifestation back to acute hepatitis. To test this hypothesis, mice were infected with increasing doses of RVFVmiR-122 or RVFVmiR-184 ranging from 2 to 200,000 TCID_50_. Most RVFVmiR-184-infected mice succumbed to disease between 3–6 dpi, with highest viral RNA loads in the liver, consistent with acute hepatitis (Fig. 3A and B). Two RVFVmiR-184-infected mice survived until 9 and 15 dpi, respectively, and these mice had the highest viral RNA loads in the brain (Fig. 3A, closed triangles). In contrast, mice infected with different doses of RVFVmiR-122 all survived the early stage (Fig. 3A). The mice that succumbed to disease showed clinical signs of CNS disease with highest viral RNA loads in the brain (Fig. 3C, closed circles). Unexpectedly, three out of five mice in both 2,000 and 200,000 TCID_50_ groups survived until the end of the experiment (27 dpi), with low to undetectable levels of viral RNA in the liver, brain, and spleen tissues at the time of terminal euthanasia (Fig. 3C, open circles). Surprisingly, not only did increased dose of RVFVmiR-122 not overcome the effect of miR-122-mediated suppression, but it also led to increased survival, leading us to hypothesize that higher doses of RVFVmiR-122 triggered stronger innate immune responses which ultimately protected mice from lethality.

### Mice infected with RVFVmiR-122 had self-limiting liver disease due to restricted viral replication in the liver

**Fig 4:**
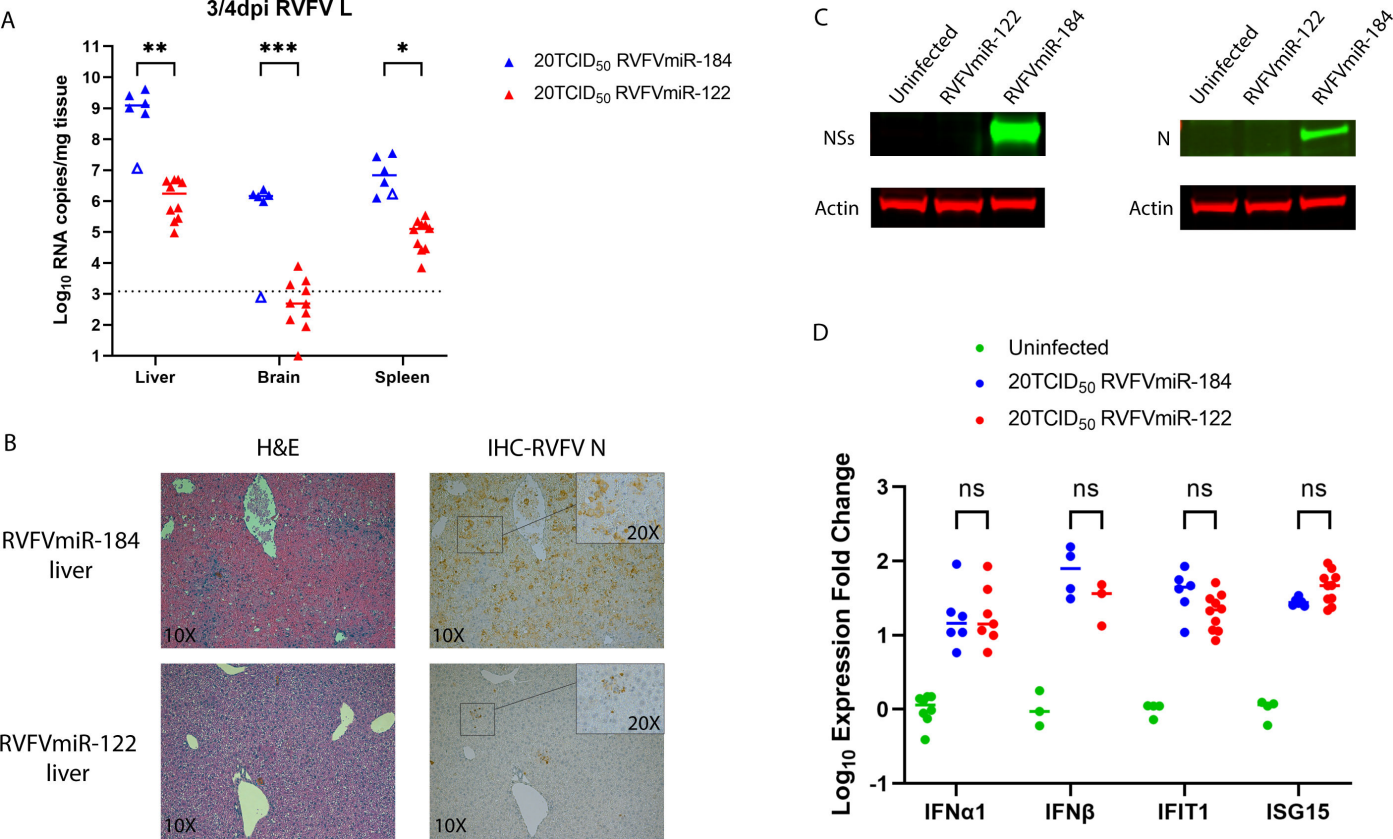
Mice infected with RVFVmiR-122 had self-limiting liver disease due to restricted viral replication in the liver. (**A**) Viral RNA loads in the liver, brain, and spleen of mice infected with either RVFVmiR-184 (*n* = 6) or RVFVmiR-122 (*n* = 10) at 3 or 4 dpi. Open triangle represents one mouse euthanized at 4 dpi without showing any symptoms. Dotted line indicates LOD of qRT-PCR. Multiple unpaired *t*-tests were performed for statistical analysis. *P < 0.05, **P < 0.01, ***P < 0.001. (**B**) Examples of liver tissue staining by H&E or IHC probing for RVFV N protein imaged with 10× objective. Insets of indicated areas were imaged with 20× objective. (**C**) Western blots of liver tissue homogenates from mice either uninfected or infected with RVFVmiR-184 or RVFVmiR-122 probing for RVFV proteins and actin. (**D**) Levels of selected IFNs and ISGs in the liver tissues of mice euthanized in (**A**) were measured by RT followed by qPCR and normalized to GAPDH control gene. Expression fold change of each IFN or ISG in infected mice was normalized to the mean of expression fold change of that IFN or ISG in uninfected mice.

Since the survival study and the dose titration study collected samples from infected mice at the time of terminal euthanasia, we had not assessed disease at earlier time points. Based on the restricted replication of RVFVmiR-122 in primary mouse hepatocytes and the survival of RVFVmiR-122-infected mice from acute hepatitis, we hypothesized that RVFVmiR-122 had restricted replication in the liver and that infected mice were able to clear the virus from the liver over time. To test this hypothesis, we infected mice with either RVFVmiR-184 or RVFVmiR-122 at 20 TCID_50_, the dose at which consistent phenotypes were observed: all RVFVmiR-184-infected mice died of acute hepatitis and all RVFVmiR-122-infected mice died of late-onset encephalitis (Fig. 3A). We initially planned a scheduled 4 dpi euthanasia for this experiment but had to euthanize some mice at 3 dpi as they met euthanasia criteria [as previously described, (27)]. RVFVmiR-122-infected mice had approximately 3–4 log less viral RNA in the liver and brain compared to those infected with RVFVmiR-184 at 3–4 dpi (Fig. 4A). Consistent with the viral RNA data, RVFV-specific IHC revealed restricted viral spread within the liver in RVFVmiR-122-infected mice (Fig. 4B). Additionally, levels of RVFV-specific proteins NSs and N were undetectable in the liver tissues of mice infected with RVFVmiR-122, supporting that the change in disease manifestation is due to restricted hepatocellular replication of RVFVmiR-122 (Fig. 4C). Previous studies have demonstrated that the NSs protein is an innate antagonist by directly preventing the interaction between SAP30 and the transcription factor Yin Yang 1 (YY1) on the promoter of interferon-β (IFN-β), thus blocking the subsequent induction of IFN-β (23, 28). Therefore, we examined if the decrease in NSs protein level in the liver of mice infected with RVFVmiR-122 was correlated with an induction of IFN-β. To our surprise, although both RVFVmiR-122- and RVFVmiR-184-infected mice showed an increase in IFN-β level compared to uninfected mice, there was no significant difference in levels of IFNα1, IFN-β, or two downstream interferon-stimulated genes (ISGs), interferon-induced proteins with tetratricopeptide repeats 1 (IFIT1) and ISG15, between the two infected groups at 3–4 dpi, measured by unpaired *t*-tests (Fig. 4D). These data show self-limiting liver disease in RVFVmiR-122-infected mice, secondary to restricted hepatocellular replication.

### The difference in disease manifestation observed in RVFVmiR-122-infected mice was eliminated in Mir-122 KO mice

**Fig 5:**
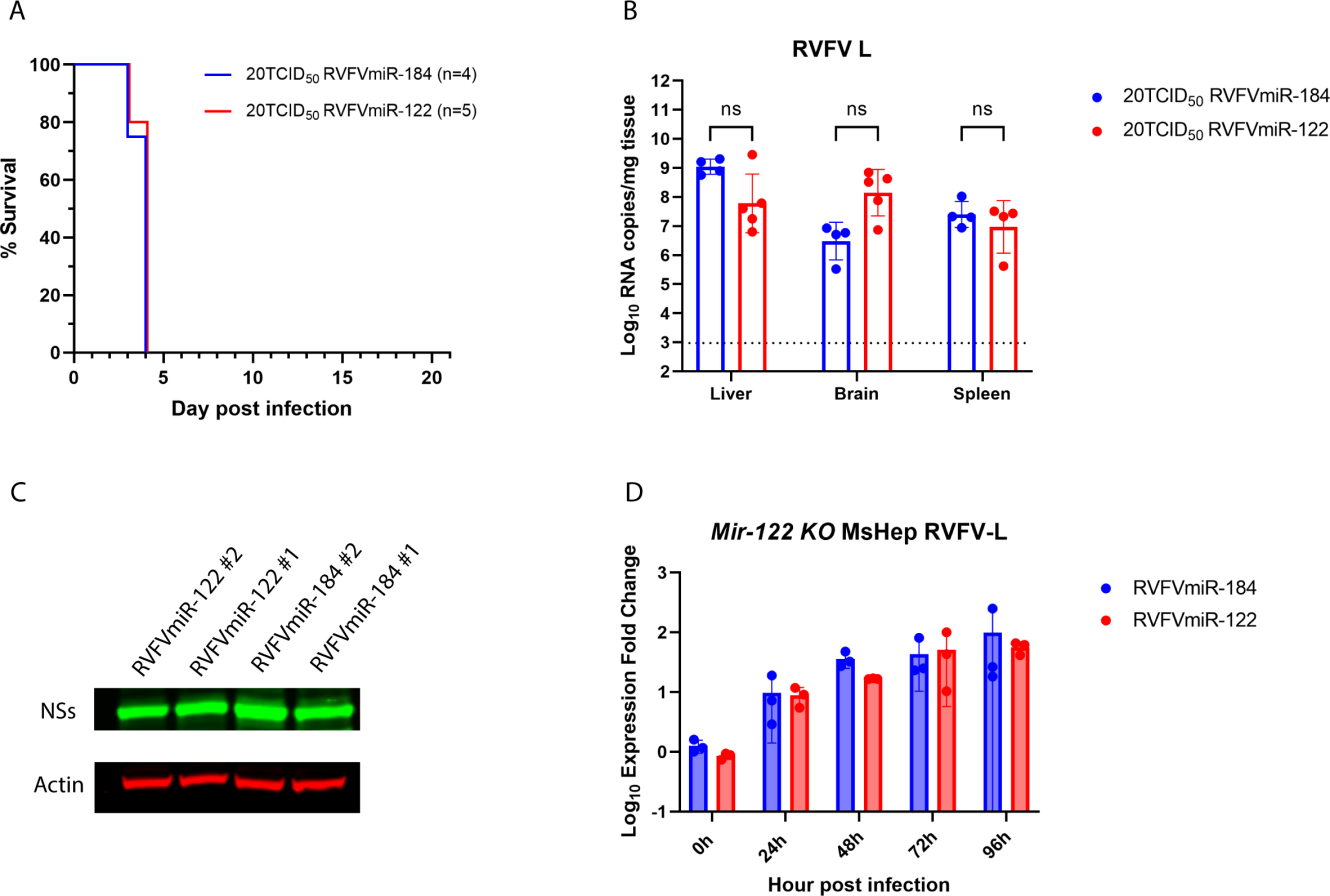
The change in disease manifestation in C57BL/6 mice infected with RVFVmiR-122 was eliminated in *Mir-122 KO* mice. (**A**) *Mir-122 KO* mice were monitored for survival after footpad infection with either RVFVmiR-184 or RVFVmiR-122. Number of mice in each group is indicated. (**B**) Viral RNA loads of RVFV L segment in the liver, brain, and spleen tissues from mice at the time of terminal euthanasia. Dotted line indicates LOD of qRT-PCR. Multiple unpaired *t*-tests were performed for statistical analysis. (**C**) Western blots of liver tissue homogenates from representatives of *Mir-122 KO* mice infected with RVFVmiR-184 or RVFVmiR-122 probing for RVFV NSs protein and actin. (**D**) Primary mouse hepatocytes isolated from *Mir-122 KO* mouse were seeded and infected with either RVFVmiR-184 or RVFVmiR-122 at an MOI of 0.1. RNA was extracted from cells in triplicate and used for qRT-PCR targeting RVFV L or GAPDH as control gene. Fold expression change of RVFV L relative to GAPDH at each time point was normalized to that at 0 h.

To confirm that the specificity of the change in disease manifestation in RVFVmiR-122-infected mice was dependent upon the expression of miR-122, we infected *Mir-122 KO* mice with either RVFVmiR-184 or RVFVmiR-122 at a dose of 20 TCID_50_. As expected, RVFVmiR-122-infected mice succumbed to disease 3–4 dpi, which was similar to control mice infected with RVFVmiR-184 (Fig. 5A). Statistical analysis using unpaired *t*-tests found no significant difference in viral RNA loads in the liver, brain, or spleen between these two groups of infected mice (Fig. 5B). The level of RVFV-specific protein in the liver of RVFVmiR-122-infected mice was the same as that in RVFVmiR-184-infected mice (Fig. 5C). Additionally, primary mouse hepatocytes isolated from *Mir-122 KO* mouse did not show a difference in viral RNA over time after infection between RVFVmiR-184 or RVFVmiR-122 (Fig. 5D). The difference in disease manifestation observed for RVFVmiR-122 was eliminated in mice without expression of miR-122, demonstrating the specificity of the phenotype.

### Faster clearance of the virus was observed in mice infected with higher doses of RVFVmiR-122

**Fig 6:**
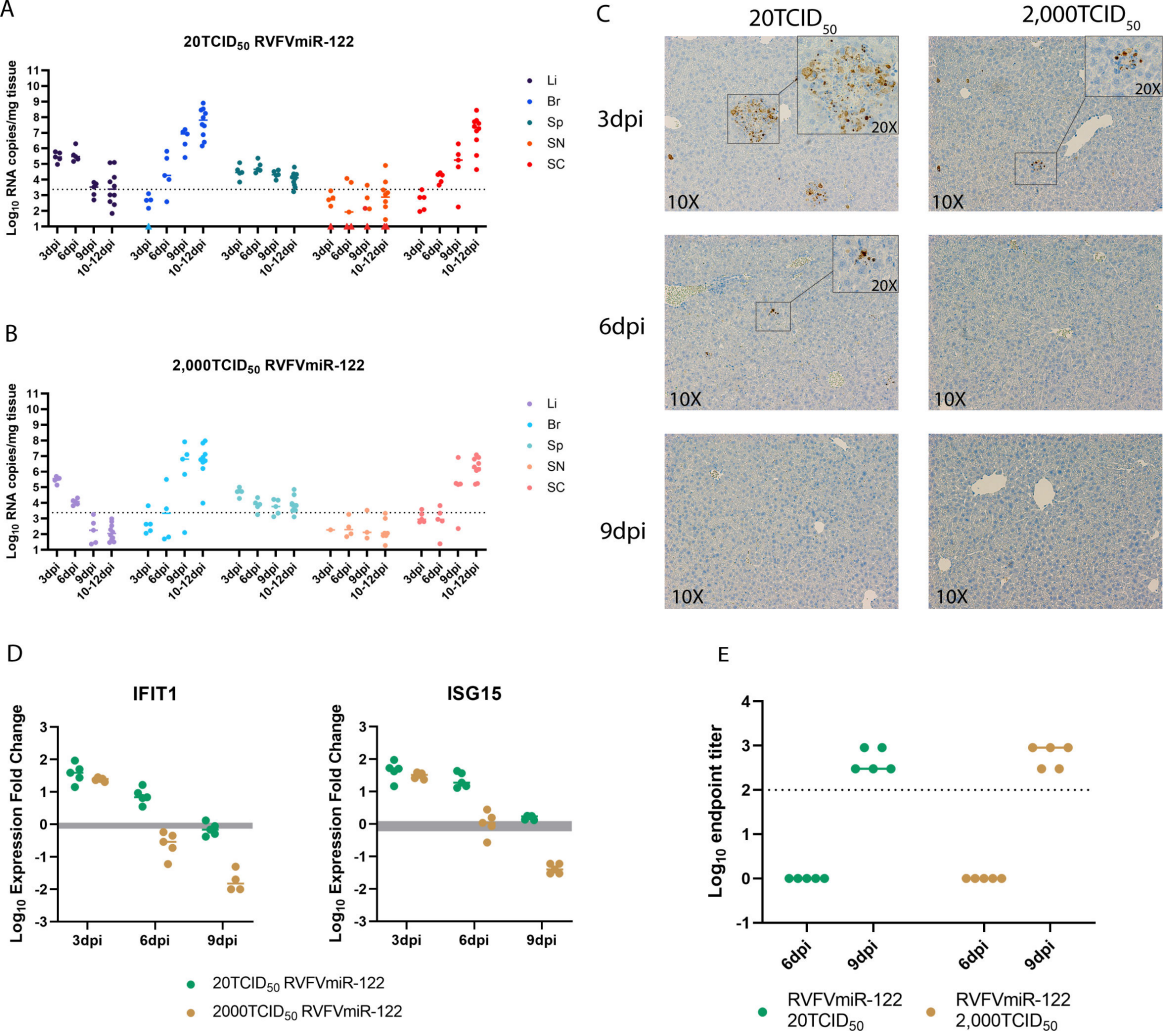
Mice infected with a higher dose of RVFVmiR-122 were able to clear the virus from the liver faster than those infected at a lower dose. (**A** and **B**) Viral RNA loads in the liver (Li), brain (Br), spleen (Sp), sciatic nerve (SN), and spinal cord (SC) of mice infected with RVFVmiR-122 at either 20 TCID_50_ (**A**) or 2,000 TCID_50_ (**B**) at 3 dpi (*n* = 5), 6 dpi (*n* = 5), 9 dpi (*n* = 5), and 10–12 dpi (*n* = 10). Dotted line indicates LOD of qRT-PCR. (**C**) Examples of liver tissue staining by IHC probing for RVFN N protein imaged with 10× objective. Insets of indicated areas were imaged with 20× objective. (**D**) Levels of IFIT1 and ISG15 in the liver tissues of mice euthanized at 3, 6, or 9 dpi were measured by RT followed by qPCR and normalized to GAPDH control gene. Gray area indicates the expression fold change of each ISG normalized to GAPDH in uninfected mice. Line indicates the median in each group. (**E**) Antibody titers at 6 dpi and 9 dpi in the serum of mice infected with RVFVmiR-122 at 20 TCID_50_ or 2,000 TCID_50_. Solid line indicates the median in each group. Dotted line indicates LOD.

During the initial dose titration experiment, 60% of mice infected with RVFVmiR-122 at higher doses (2,000 TCID_50_ and 200,000 TCID_50_) survived until the end of the experiment (Fig. 3A). As this was not expected, we conducted a serial euthanasia study to compare the disease progression of mice infected with RVFVmiR-122 at 20 TCID_50_ and 2,000 TCID_50_. Groups of mice underwent scheduled euthanasia at 3, 6, and 9 dpi. Euthanasia was required for a portion of mice due to clinical disease between 10 and 12 dpi, so these animals were included with mice euthanized at 12 dpi as scheduled; all but one of these mice demonstrated clinical symptoms. Mice infected with either dose had a decrease in viral RNA load in the liver and an increase in viral RNA load in the brain over time, which was consistent with their disease manifestation (Fig. 6A and B). However, whereas the level of viral RNA in the liver of mice infected with 20 TCID_50_ RVFVmiR-122 remained the same at 6 dpi as that at 3 dpi (*P* = 0.5015), mice infected with 2,000 TCID_50_ had viral RNA significantly lower at 6 dpi compared to that at 3 dpi (*P* < 0.0001) analyzed by one-way ANOVA, suggesting that mice infected at higher dose of RVFVmiR-122 were able to clear the virus in the liver at a faster rate (Fig. 6A and B). This was confirmed by RVFV-specific IHC data, in which no antigen staining was detected in the liver of mice infected with 2,000 TCID_50_ RVFVmiR-122 as early as 6 dpi, whereas mice infected with 20 TCID_50_ RVFVmiR-122 still showed antigen staining until 9 dpi in the liver (Fig. 6C). Viral RNA in both brain and spinal cord of infected mice increased over time in both groups, consistent with the clinical manifestation of CNS disease (Fig. 6A and B). We hypothesized that the faster clearance of RVFVmiR-122 from the liver at the higher infection dose might be due to a stronger innate or adaptive immune response. However, we did not observe a difference in IFIT1 or ISG15 expression at 3 dpi for mice infected with RVFVmiR-122 at 20 or 2,000 TCID_50_, nor did we see a difference in RVFV-specific antibody titer at 6 or 9 dpi for mice infected at the two different doses (Fig. 6D and E). Lower levels of IFIT1 and ISG15 at later time points in mice infected with RVFVmiR-122 at 2,000 TCID_50_ compared to those infected at 20 TCID_50_ likely reflect lower levels of viral RNA in the liver to stimulate innate viral detection (Fig. 6D). Altogether, these data supported that mice infected with RVFVmiR-122 at higher doses can clear the virus at a faster rate, although the underlying mechanism remains to be determined.

## DISCUSSION

Since the liver is a key and major target of RVFV, defining the contribution of hepatocyte tropism to RVFV pathogenesis is crucial to understanding disease manifestations and can provide important insights into prevention and treatment of RVFV disease. Canonical ways to evaluate the effects of cellular tropism on viral pathogenesis regarding individual cell types include modifications of either the host or the virus. As hepatocytes are critical to life, they cannot be depleted from the host. Removal of the virus receptor on the host cell surface was not an option for a long time as no definitive receptor had been identified for RVFV until recently (29). With regard to virus manipulation, the traditional approach of using an attenuated virus with less pathogenesis would limit our studies. Here, we constructed recombinant viruses with the goal of restricting replication in the liver to dissect the relationship between hepatocyte tropism and pathogenesis of RVFV. We demonstrate that incorporating sequences targeted by hepatocyte-specific miR-122 into the RVFV genome caused restricted viral replication in the liver and altered disease manifestations in an RVFV acute hepatitis mouse model.

MicroRNA-122 is a key player in liver biology and disease. It is highly expressed in the liver, where it constitutes 70% of the total miRNA pool, and is absent from most other tissues (19, 21). In primary mouse hepatocytes that express miR-122, RVFVmiR-122 showed significantly lower viral RNA titers at 72 h and 96 h. It might seem counterintuitive that a 1-log change *in vitro* would have such a great impact *in vivo* and completely reroute the disease manifestation of C57BL/6 mice after infection. This could be due to the dramatic decrease of the levels of miR-122 in primary mouse hepatocytes after *ex vivo* plating (30), or infection itself could alter miR-122 expression. However, due to the design of our recombinant viruses, assays quantifying levels of miRNA also detect sequences on transcribed (+) sense viral RNA.

Our lab has previously demonstrated that C57BL/6 mice infected with WT RVFV at a dose as low as 0.2 TCID_50_ die of acute hepatitis 3–5 dpi (27). In this study we generated RVFVmiR-184 as a control virus. MiR-184 is one of the most highly expressed miRNAs in *Aedes* and *Culex*, which are two mosquito vectors associated with RVFV transmission in various parts of Africa (17, 26, 31). In humans, miR-184 has been identified in oligodendrocyte progenitor cells where it helps promote oligodendrocyte differentiation (32). No presence of miR-184 in the liver has been reported (20). Our results showed that C57BL/6 mice infected with RVFVmiR-184 at various doses started to show clinical symptoms and required euthanasia at 3–5 dpi, consistent with WT RVFV-infected mice. There were two RVFVmiR-184-infected mice that lived until 9 dpi or 15 dpi and died of CNS disease, suggesting RVFVmiR-184 may have some low level of attenuation compared to WT RVFV. Nonetheless, RVFVmiR-184 served as a better control than WT RVFV as it controls for the impact of sequence incorporation into the viral genome on viral pathogenesis.

In our dose titration study, we expected that mice infected with increased doses of RVFVmiR-122 would eventually overcome the capacity of miR-122 expressed in the liver and change the disease manifestation back to acute liver disease. Surprisingly, not only did the mice that succumbed to disease die of late-onset encephalitis, but we also observed an increase in survival rate with an increase in dose, suggesting protection via an enhanced immune response triggered by detection of increased amounts of viral RNA. The binding of miR-122 to its target sequences on RVFVmiR-122 could create a double-stranded RNA intermediate that could be sensed by retinoic acid-inducible gene I (RIG-I), an RNA helicase receptor that recognizes dsRNAs. This recognition activates an adaptor protein mitochondrial antiviral-signaling protein (MAVS) and triggers downstream activation of IFN-β and subsequent ISGs (33, 34). Stimulation of several ISGs have been implicated upon RVFV infection, including IFITs and ISG15 (35, 36). However, we did not detect a difference in IFIT1 or ISG15 at 3 dpi in the liver of RVFVmiR-122-infected mice compared to control mice. We did observe a faster clearance of virus from the liver over time in mice infected with the higher dose of RVFVmiR-122, which correlated with a faster return of IFIT1 and ISG15 to the normal level. These two observations could occur because of an earlier induction of IFN-β which had already normalized by 3 dpi when we measured it. In the future, mice infected with RVFVmiR-122 could be euthanized at 12 h or 18 h to rigorously examine the innate immune response involving RIG-I and MAVS at earlier times post-infection. Alternatively, increasing evidence suggests another viral RNA sensor, protein kinase R (PKR), plays a critical role in IFN induction upon viral infection (37, 38). Degradation of PKR is promoted by NSs and essential for RVFV infection (39, 40). Future studies will investigate the underlying mechanisms controlling the survival of mice at higher doses of RVFVmiR-122.

In our study, C57BL/6 mice infected with RVFVmiR-122 consistently developed late-onset encephalitis and at higher doses, some even survived infection. Patients who developed CNS disease after RVFV infection have high mortality rates, necessitating the importance of studying this disease manifestation (8). However, traditional mouse models of RVFV encephalitis rely on either the mice to be immunosuppressed and infected with an attenuated virus or administration of virus via intranasal or aerosol exposure (41, 42). Yet, this does not mimic the natural route of infection via mosquito bite in a healthy human. Our lab has recently characterized a Collaborative Cross mouse strain, CC057, as a new model to study RVFV encephalitis via footpad (FP) infection (43). CC057 mice were demonstrated to be resistant to acute hepatitis caused by RVFV and consistently developed late-onset encephalitis, implicating a role for host genetics in RVFV disease. In contrast, in this study, we modified the virus instead of the host and established a new model to study RVFV encephalitis in C57BL/6, a mouse strain that traditionally serves as a lethal acute hepatitis model for RVFV. A major advantage of using RVFVmiR-122 in C57BL/6 mice over WT RVFV in CC057 mice for studies of RVFV encephalitis is the availability of reagents and genetically modified mouse strains to enable mechanistic studies. Importantly, both models use footpad injection which mimics a peripheral route of exposure as seen in natural exposure to mosquito bite. These two models can greatly complement each other in future studies. For example, it remains to be characterized how RVFV enters the brain. In the CC057 model, viral trafficking via the sciatic nerve did not appear to be a convincing route for CNS entry (43). In our study, viral RNA level was also low in the sciatic nerve over time after RVFVmiR-122 infection, again suggesting that nerve trafficking is not the mode of viral entry into the brain for RVFV. Both of these RVFV encephalitis models will enable studies to assess other possible routes of virus entry into the brain.

In conclusion, this study utilized miRNA-targeted viruses to evaluate the role of viral replication in hepatocytes in RVFV pathogenesis and demonstrated that restricting viral replication in hepatocytes altered disease outcome. Techniques used in this study provide an innovative tool to the RVFV field that could be applied to study the consequences of limiting RVFV replication in other target cells.

## MATERIALS AND METHODS

### Generation, growth, and titer of recombinant viruses RVFVmiR-122 and RVFVmiR-184

All recombinant viruses were generated using the published RVFV reverse-genetics system (44), which was kindly shared with us by Dr. César Albariño (Center for Disease Control, United States). To facilitate the cloning of miRNA-targeted sequences into the RVFV S segment rescue plasmid, a 16 bp insertion (5´-gagctccacatatgtg-3´) was placed in the intergenic region after the NSs coding region, corresponding to between bp 833 and 834 of the RVFV S segment (GenBank: DQ380149.1). This insertion provided AleI and SacI restriction sites in the S segment rescue plasmid, referred to as WTAleSac S segment. miR target sequences were generated commercially and consisted of four repeats of each miR; a 4 bp repeat of CGAT was inserted between miR repeats 1–2 and 3–4. A KpnI site was inserted between miR repeats 2–3 to facilitate screening of clones. The cassette was flanked by EcoRV sites for blunt ended cloning into the AleI site of the WTAleSac S segment rescue plasmid. Clones were selected such that miR targets are in the minus sense (genome) segment. Clones were confirmed by Sanger sequencing and then recombinant RVFVmiR-122 and RVFVmiR-184 were rescued by transfection into BSRT7 cells [kindly provided by Ursula Buchholz, NIAID, and initially described in reference (45)] as previously described (44). Each virus was passaged twice and then sequence confirmed using lllumina sequencing. Viral titers of passage two stocks were determined using a focus forming unit (FFU) assay. Briefly, virus was serially diluted in duplicate in Dulbecco’s Modified Eagle Medium with 10% fetal bovine serum (DMEM-10) and then added to 96-well plates of Vero E6 cells seeded the day before at a density of 1 × 10^4^ cells per well. Cells were incubated at 37°C for 1 h followed by removal of inoculum and replacement with 1.5% carboxymethyl cellulose in MEM-10 for 18 h. Cells were then washed in phosphate buffered saline (PBS), fixed in 10% formalin and immunostained using a custom rabbit polyclonal anti-RVFV N primary antibody [GenScript, 1:1,000 dilution in 5% non-fat milk in PBS-0.1% Tween 20 (PBST)] followed by an horseradish peroxidase (HRP)-conjugated donkey anti-rabbit secondary antibody (Jackson ImmunoResearch #711-035-152, 1:1,000 dilution in 5% non-fat milk-PBST). Plates were scanned and foci were counted using a CTL ImmunoSpot Analyzer to calculate FFU per milliliter for each virus.

### *In vitro* viral replication in cell lines or primary hepatocytes

Primary hepatocytes isolated from C57BL/6 or *Mir-122* whole-body knockout mice (*Mir-122 KO*) using a two-step collagenase perfusion (46) were seeded at 1.5 × 10^5^ viable cells per well in 24-well Primaria Cell Culture plates (Corning) in seeding media: DMEM-F12 with 15 mM HEPES (Corning), 5% fetal bovine serum (FBS) (Atlanta Biologicals, Atlanta, GA), and Antibiotic-Antimycotic Solution (Corning). After 4 hrs, seeding media were replaced with maintenance media: DMEM-F12 with 15 mM HEPES (Corning), 0.5% FBS (Atlanta Biologicals), Antibiotic-Antimycotic Solution (Corning), and ITS Supplement (containing 1 µg/mL insulin, 0.55 µg/mL transferrin, and 0.67 ng/mL sodium selenite; Roche). Vero E6 (in DMEM-10) and HepG2 (in MEM-10) cells were seeded on 24-well plates at 1 × 10^5^ cells per well. After 16–24 h of plating, cells were infected with either RVFVmiR-184 or RVFVmiR-122 (MOI = 0.1) for 1 h at 37°C, washed once with PBS, and incubated with fresh medium. Supernatants were collected at various times post-infection and used for FFU assay as described above.

### Design of mouse study

All C57BL/6J mice used in this study were 6- to 8-week-old females and purchased from Jackson Laboratory (#000664). *Mir-122 KO* were obtained from Dr. Kalpana Ghoshal at Ohio State University (47) and bred and maintained at University of Pittsburgh. All mice were housed in HEPA filtration racks at the RBL’s ABSL-3 facility and provided *ad libitum* access to food and water. Mice were infected with RVFVmiR-184 or RVFVmiR-122 under isoflurane anesthesia via left-rear FP injection. Viral infection doses in these studies ranged from 2 to 200,000 TCID_50_ per animal. Mice received a 20 µL injection of virus diluted in sterile PBS. For all experiments, daily weights were recorded, and mice were evaluated daily for clinical signs of disease. For survival studies, mice were euthanized according to a predetermined clinical scoring method (27). For the serial euthanasia study, mice were euthanized on a specific day post-infection. At the time of euthanasia, mice were anesthetized with isoflurane and blood was collected via cardiac puncture. Following cervical dislocation, liver, spleen, brain, and where applicable, sciatic nerve, (left leg) and spinal cord were collected in sterile PBS supplemented with 1× Antibiotic-Antimycotic (Gibco #15240062). Blood samples were analyzed using a VETSCAN HM5 hematology analyzer (Abaxis) or a VETSCAN VS2 chemistry analyzer (Abaxis) with the Mammalian Liver Profile reagent rotor. Tissue samples were weighed and then homogenized using a D2400 Homogenizer (Benchmark Scientific) for subsequent RNA extraction and viral RNA load quantitation.

### RNA extraction and qRT-PCR

RNA was extracted from tissue samples using TRIzol reagent (Invitrogen #15596026) followed by Direct-zol RNA Miniprep Kits (Zymo #R2052). Samples for qRT-PCR targeting the RVFV L or S segment were prepared with the Reliance One-Step Multiplex Supermix (BioRad #12010221). Reactions were performed using a C1000 Touch Thermal Cycler/CFX96 Real-Time System (Bio-Rad) with the following conditions: 50°C for 15 min, 95°C for 3 min, and then 40 cycles of 95°C for 15 s and 55°C for 1 min. RVFV L or S RNA template of known quantity was serially diluted to generate a RVFV RNA standard curve. RVFV L RNA template was generated as previously described (27). RVFV S RNA template was generated from a WT RVFV S segment rescue plasmid which was digested with BglII (NEB) to create a linear template for use in a TranscriptAid T7 High Yield *in vitro* transcription reaction (Thermofisher #K0441). Resultant RNA was purified with the GeneJET RNA Purification kit (Thermofisher #K0702), diluted to known copies per milliliter in RNase-free water for use as qRT-PCR standard curve template. RNA copies for each experimental sample were normalized by tissue weight. The limit of detection of this assay was calculated using the highest *Cq* value detected in the standard curve multiplied by dilution factor and then divided by the average weight of all sampled tissues.

RNA extracted from Vero E6, HepG2, or primary mouse hepatocytes was used for reverse transcription with LunaScript^®^ RT Master Mix Kit (Primer-free NEB #E3025) and RT primer for miR-122 or U6 (ThermoFisher Scientific, predesigned TaqMan MicroRNA Assays ID# 002245 and 001973) to generate cDNA using a MiniAmp Plus Thermal Cycler (Applied Biosystems) with the following reactions: 16°C for 30min, 55°C for 10min, and then 95°C for 1min. Generated cDNA was used for qPCR with TaqMan MicroRNA Assays for miR-122 or U6. Q-PCR was run on a C1000 Touch Thermo Cycler/CFX96 Real-Time System (Bio-Rad) with the following conditions: 94°C for 2 min, 94°C for 15 sec, and then 40 cycles of 55°C for 15 sec and 68°C for 15 sec.

### Histopathology

Liver and brain tissues were fixed in 10% formalin, paraffin embedded, and sectioned using standard methods. Tissues were either stained with H&E or processed with an IHC assay through the Pitt Biospecimen Core. Tissues were evaluated for anti-RVFV immunoreactivity using a custom polyclonal rabbit anti-RVFV N protein antibody (Genscript, 1:200).

### Western blot

Liver tissue samples were homogenized in Laemmli buffer (20 mg/500 µL) and then analyzed by Western blot. Samples were loaded on 12% SDS-PAGE gels (Invitrogen #XP00122BOX) which were run at 200 V for 45 min and then transferred to nitrocellulose membrane (Invitrogen #LC2000) at 10 V for 1 h. Membranes were blocked with 5% BSA-PBST for 1 h at room temperature and probed using either RVFV N (Genscript, rabbit polyclonal, 1:2,000), or RVFV NSs (Genscript, rabbit polyclonal, 1:2,000), in addition to actin (Invitrogen #MA-11869, mouse monoclonal, 1:2,000) as a loading control. After three washes with PBST, membranes were incubated for 1 h at room temperature with corresponding secondary antibodies (IRDye 800CW Donkey anti-rabbit, 926-32213, 1:20,000; IRDye 680RD anti-mouse, 926-68072, 1:20,000). After another three washes with PBST, membranes were imaged using Li-Cor Odyssey CLx Imager.

### Reverse transcription and qPCR of host genes

RNA extracted from liver tissues of infected mice was used for reverse transcription with iScript Reverse Transcription Supermix (BioRad #1708841) to generate cDNA using a MiniAmp Plus Thermal Cycler (Applied Biosystems) with the following reactions: 25°C for 5 min, 46°C for 20 min, and then 95°C for 1 min. Generated cDNA was used for qPCR with iTaq Universal SYBR Green Supermix (BioRad #1725121) and PrimePCR assays for IFNα1, IFNβ1, IFIT1, or ISG15 (BioRad #10025636). qPCR was run on a C1000 Touch Thermo Cycler/CFX96 Real-Time System (Bio-Rad) with the following conditions: 94°C for 2 min, 94°C for 15 s, and then 40 cycles of 55°C for 15 s and 68°C for 15 s.

### Enzyme-linked immunosorbent assay (ELISA)

MaxiSorp plates (Thermo Scientific) were coated with 1:1,000 diluted lysate in PBS from either uninfected or DelNSm/DelNSs RVFV-infected Vero E6 cells and left at 4°C overnight. Plates were incubated with blocking buffer (5% non-fat milk in PBST) at 37°C for 1 h. Mouse serum samples collected at the time of euthanasia were serially diluted in blocking buffer and then incubated on plates in duplicate at 37°C for 2 h. Uninfected mouse serum was included on each plate as a negative control. Following serum incubation, plates were washed three times with PBST, and then incubated at 37°C with HRP-conjugated donkey anti-mouse IgG (Jackson ImmunoResearch #715-035-150) at 1:5,000 in blocking buffer for 1 h. Plates were then washed three times with PBST and incubated in tetramethylbenzidine (TMB) substrate (SeraCara #5120-0047), followed by TMB stop solution. Optical density (OD) at 450 nm was measured using a Biotek plate Reader. Data were analyzed in Excel by subtracting values of uninfected lysate from values of the RVFV-infected lysate plate. The endpoint titer was defined as the highest dilution of serum that resulted in an OD value at least three standard deviations above the average obtained from all negative mouse serum control wells on the same plate.

### Statistics

GraphPad Prism 9 was used for graph generation and statistical analysis. Data of qRT-PCR, qPCR, and ELISA were analyzed in Excel. Statistical tests for indicated data set are specified in the text or figure legends.
